# Absence of Complementary Sex Determination in the Parasitoid Wasp Genus *Asobara* (Hymenoptera: Braconidae)

**DOI:** 10.1371/journal.pone.0060459

**Published:** 2013-04-02

**Authors:** Wen-Juan Ma, Bram Kuijper, Jetske G. de Boer, Louis van de Zande, Leo W. Beukeboom, Bregje Wertheim, Bart A. Pannebakker

**Affiliations:** 1 Evolutionary Genetics, Centre for Ecological and Evolutionary Studies, University of Groningen, Groningen, The Netherlands; 2 Theoretical Biology, Centre for Ecological and Evolutionary Studies, University of Groningen, Groningen, The Netherlands; 3 Behaviour & Evolution Group, Department of Zoology, University of Cambridge, Cambridge, United Kingdom; 4 Laboratory of Entomology, Wageningen University, Wageningen, The Netherlands; 5 Laboratory of Genetics, Wageningen University, Wageningen, The Netherlands; VIB & Katholieke Universiteit Leuven, Belgium

## Abstract

An attractive way to improve our understanding of sex determination evolution is to study the underlying mechanisms in closely related species and in a phylogenetic perspective. Hymenopterans are well suited owing to the diverse sex determination mechanisms, including different types of Complementary Sex Determination (CSD) and maternal control sex determination. We investigated different types of CSD in four species within the braconid wasp genus *Asobara* that exhibit diverse life-history traits. Nine to thirteen generations of inbreeding were monitored for diploid male production, brood size, offspring sex ratio, and pupal mortality as indicators for CSD. In addition, simulation models were developed to compare these observations to predicted patterns for multilocus CSD with up to ten loci. The inbreeding regime did not result in diploid male production, decreased brood sizes, substantially increased offspring sex ratios nor in increased pupal mortality. The simulations further allowed us to reject CSD with up to ten loci, which is a strong refutation of the multilocus CSD model. We discuss how the absence of CSD can be reconciled with the variation in life-history traits among *Asobara* species, and the ramifications for the phylogenetic distribution of sex determination mechanisms in the Hymenoptera.

## Introduction

Sexually reproducing organisms have evolved a wide diversity of mechanisms to establish the two sexes [1]–[4]_._ Examples of chromosomal sex determination systems are male or female heterogamety, haplodiploidy and multifactorial sex determination [5]–[7]. Insect sex determination systems have been relatively well studied, with a main focus on the orders of Diptera, Lepidoptera and Hymenoptera. The whole insect order Hymenoptera, comprising ants, bees, wasps and sawflies, exhibits haplodiploid reproduction, but the molecular regulation of sex determination varies. The primary signal in hymenopterans is derived from the number of chromosome sets in embryos: diploids develop into females and haploids into males [6], [8]–[11]. Thus far, two genetic mechanisms of sex determination have been empirically supported in the Hymenoptera: complementary sex determination (CSD) [6], [8], [12]–[15] and maternal control sex determination, although the latter mechanism has only been documented for the parasitoid *Nasonia* [11], [16], [17], and its preponderance among other haplodiploid species remains to be determined.

CSD has now been documented in over 60 hymenopteran species [9], [18]. Whiting [8], [12] was the first to propose that sex in some hymenopterans is determined by allelic complementation at a single locus (sl-Complementary Sex Determination or sl-CSD): heterozygosity at the *csd* locus leads to female development, whereas homozygosity or hemizygosity at the *csd* locus initiates the development of diploid or haploid males respectively. A *csd* gene was originally identified in the honeybee *Apis mellifera* and has also been documented from some bumble bees and ants, where it is a duplication of the gene *feminizer* (an ortholog of the key sex determination gene *transformer*) [15], [19], [20]. Presence of the sl-CSD phenotype is typically demonstrated by inbreeding crosses and the associated increase in homozygous diploid males compared to outcrosses. Diploid males are often sterile or inviable and constitute a considerable fitness cost [21]–[23]. One way of genetically reducing the production of diploid males is to increase the number of *csd* loci [24], i.e. multilocus CSD (ml-CSD), which was proposed by Snell [13] and Crozier [14] for species with regular but not exclusive inbreeding. Under ml-CSD, female development occurs when at least one *csd* locus is heterozygous, so that for the development of diploid males, homozygosity at all *csd* loci is required [14]. Since identification of the number of *csd* loci using molecular tools is extremely laborious in non-model systems, a more suitable alternative to identify the presence of ml-CSD is exposing populations to multiple generations of inbreeding. Successful applications of such experiments have confirmed the presence of ml-CSD in two *Cotesia* species [25], [26]. The general prevalence of ml-CSD among hymenopterans, however, remains unknown and requires experimental tests in more species [9], [18].

There is a strong link between the mode of sex determination and specific life-history traits within the Hymenoptera. For example, natural inbreeding and the corresponding mating systems are incompatible with sl-CSD, because this would dramatically increase the production of diploid males [21]–[23], [27]–[29]. Inbreeding may select for ml-CSD to avoid diploid male production, since under ml-CSD homozygosity is required at a larger number of sex loci, genetically reducing diploid male production. Although the number of tested species is still low, the various sex determination mechanisms in the genus *Cotesia* (Hymenoptera, Braconidae) [25], [26], [28], [30]–[32] suggest a link between inbreeding levels and presence of CSD types. *Asobara* is another braconid parasitoid genus which exhibits substantial diversity in life-history traits. They are solitary larval endoparasitoids of various *Drosophila* species [33] that have an aggregated larval distribution [34]. This type of host distribution allows a single *Asobara* female to produce multiple offspring near each other, thus resembling gregariousness and allowing for sibmating. Interestingly, species-specific dispersal patterns, such as the patch-defense behavior displayed by *A. citri* females during oviposition, and aggregated host-searching behavior in *A. tabida* [35], may also contribute to differences in inbreeding levels among *Asobara* species. *Asobara tabida* occurs all over Europe and North America [36], *A. japonica* is limited to Japan [37], *A. citri* occurs in Africa [38], and *A. pleuralis* is mainly found in South-East Asia [39]. Taken together, these aspects make this genus an interesting candidate to investigate the presence of CSD types.

Beukeboom et al. [27] previously concluded that sl-CSD is absent in *Asobara tabida*. However, Asplen et al. [18] hypothesized that ml-CSD is likely present in this species based on the phylogenetic distribution of CSD in the Hymenoptera. Knowledge of the sex determination mechanism(s) in the *Asobara* genus is of key importance for several reasons. It yields more insight in the evolution of sex determination diversity at different taxonomic levels, including closely related species within a genus, in the Hymenoptera order, and in insects in general [18]. Moreover, knowledge of sex determination is essential for understanding the evolution and constraints of adaptive sex allocation [40]–[42] and for conservation management of declining populations of pollinating hymenopterans [10], [23], [25]. Here, as the first step towards elucidating the variation in sex determination mechanisms in the *Asobara* genus, we investigate the possible existence of sl-CSD and ml-CSD in four *Asobara* species. CSD is assessed by inbreeding experiments, in which consecutive generations with increasing levels of inbreeding are compared for differences in diploid male production, brood size, offspring sex ratio and pupal mortality. In addition, for a proper assessment of ml-CSD and a realistic estimate of the number of sex loci involved, formal models are essential to provide expected patterns of diploid male production and offspring sex ratios (proportion male offspring) over generations of inbreeding [6], [24]–[26].

## Materials and Methods

### Wasp Culturing

Four *Asobara* species, *A. tabida*, *A. japonica*, *A. citri* and *A. pleuralis* were collected from their native distribution ranges by third parties several years ago, and cultured in the laboratory on second instar *Drosophila* larvae as hosts at 12L: 12D and a relative humidity of 50–60%. Detailed information on strains origins, host species, and rearing temperatures is given in the supporting information, Table S1. All four *Asobara* species used in our experiments were obtained from J.J.M. van Alphen (Leiden University, The Netherlands) in 2009, and had been cultured in the laboratory for a long time.

### CSD Assay

The presence of CSD in parasitoids is generally assessed by multiple generations of inbreeding, during which the diploid male production, brood size, offspring sex ratio and pupal mortality are compared [25], [30]. Brood size, offspring sex ratio and pupal mortality are monitored because diploid males may be inviable and therefore affect brood sex ratio primarily through a loss of part of the brood. For all four *Asobara* species, the inbreeding assay started with a mother-son (M-S) cross which resulted in a maximum of two different alleles per putative sex locus, followed by multiple generations of brother-sister (B-S) crosses. Under sl-CSD, half of the fertilized eggs will be homozygous at the sex locus in an M-S cross, which will directly lead to the development of diploid males. In B-S crosses, the development of diploid males depends on whether the brother and sister share an identical *csd* allele (matched mating) or not (unmatched mating). Under ml-CSD, diploid males are only expected when all sex loci are homozygous. Therefore, under sl-CSD, half of the fertilized eggs will develop as diploid males in both M-S and B-S crosses, and the proportion of diploid males is predicted to remain 0.5 over subsequent generations of inbreeding. Under ml-CSD, the proportion of diploid males from an M-S cross is a function of the number of *csd* loci, and is predicted to increase rapidly over the subsequent generations of B-S crosses due to increasing proportions of matched matings.

### Inbreeding Experiment

We investigated different types of CSD following the methods outlined in de Boer et al. [25], by monitoring diploid male production, brood size, offspring sex ratio, and pupal mortality over nine to thirteen successive inbreeding generations for four tested *Asobara* species. We started with an outcrossed generation, followed by a single M-S cross, and 8–12 generations of B-S crosses. For *A. tabida* and *A. japonica*, the outcrossed generations were started by crossing a male and a female from two different strains (N = 31, *A. tabida*; N = 14, *A. japonica*), which increases the chance of heterozygosity at each putative sex locus in the female offspring. Only one strain of each species was available, and we set up 26 mated females for *A. citri* and 21 for *A. pleuralis* from mass culture. Subsequently, one to three virgin females were collected from the offspring of each outcross replicate, and each individual female was allowed to oviposit on approximately 50 second instar *Drosophila* larvae for one or two days to produce haploid sons. The mothers were kept at 12°C while their sons developed. After emergence of the sons, each of the surviving mothers (*A. tabida*: approximately three-weeks old, *A. japonica* and *A. citri*: two-weeks old, *A. pleuralis*: ten days old) was back-crossed with one of her sons. Subsequently, B-S crosses were continued for eight (*A. tabida*), nine (*A. japonica* and *A. citri*) and twelve (*A. pleuralis*) generations. For each generation of B-S crosses, one to three virgin females were collected per family from the previous generation and mated with a single haploid brother, the ploidy of which was analyzed by flow cytometry (see below). Crosses were done in individual plastic vials (diameter 2.4 cm, height 7.5 cm) containing a layer of agar, and each couple was given honey for 24 hrs prior to oviposition. Van Alphen and Nell [43] found that experienced *Asobara* wasps can distinguish non-parasitized host larvae from parasitized larvae and mainly oviposit on the non-parasitized larvae. Compared to non-experienced females, oviposition efficiency was increased by using experienced females in our experiments, due to reduced super-parasitism (oviposition in already-parasitized hosts) and associated host mortality. Females were given oviposition experience by providing them with approximately 100 second instar larvae for two hours. For the experimental assay, 150 second instar *D. melanogaster* larvae were offered to each experienced female in a glass bottle with agar medium and a layer of 1.5 ml yeast solution (0.4 g/ml). Females were allowed to parasitize the host larvae for 24 to 36 hours. The emerging flies were counted, and the emerging wasps were anaesthetized with CO_2_, counted and sexed by scoring the presence or absence of an ovipositor, which prominently protrudes from the posterior end of the abdomen. For each *Asobara* species, brood size and offspring sex ratio were determined per replicate per generation. After all wasps had emerged, the number of black pupae (containing either dead *Drosophila* or wasps) and empty pupae (from which either *Drosophila* or *Asobara* adults had emerged) was counted to determine the pupal mortality (proportion black pupae among all pupae per replicate) as an indication for inviable diploid males and/or inviability effects due to inbreeding.

### Detection of Diploid Males

To detect the production of diploid males with inbreeding, a range of 30 to 147 males were collected per generation during the first three (M-S cross, 1^st^ and 2^nd^ generations of B-S crosses) and the last generations of inbreeding. For *A. pleuralis*, we tested the ploidy of males in the 5^th^ generation of B-S cross (instead of 1^st^ or 2^nd^ generation of B-S cross), when a higher offspring sex ratio was observed. The number of tested males per brood per generation of each *Asobara* species is listed in Table 1. Ploidy level was analyzed with flow cytometry, following methods described by de Boer et al. [32]. In short, the head of each individual male (freshly killed by freezing at −20°C) was homogenized in 500 µl Galbraith buffer, and the DNA was stained with 10 µl propidium iodide (2.5 mg/ml). The total DNA content of approximately 2500 nuclei was measured on a Coulter Epics MXL flow cytometer (Beckman Coulter, Miami, FL, USA). Two females of each species were used as diploid references. Males were classified as haploid or diploid by comparing the DNA amount histogram to the diploid reference. Histogram figures of ploidy data were produced by WinMDI 2.9 software package (The Scripps Research Institute, La Jolla, CA, USA).

**Table 1:** Number of diploid males and sample size for each brood and generation of inbreeding in *Asobara tabida*, *A. japonica*, *A. citri* and *A. pleuralis*.

Species	Generation	No. broods tested	Average no. males tested per brood	No. diploid males (total no. male samples)
*A. tabida*	M-S	12	4	0 (52)
	B-S1	17	4	0 (66)
	B-S2	16	2	0 (32)
	B-S8	6	6	0 (36)
*A. japonica*	M-S	8	6	0 (47)
	B-S1	12	4	0 (48)
	B-S2	13	4	0 (50)
	B-S8	6	6	0 (36)
*A. citri*	M-S	14	5	0 (67)
	B-S1	13	4	0 (50)
	B-S2	12	4	1 (48)
	B-S8	6	5	0 (30)
*A. pleuralis*	M-S	12	12	1 (147)
	B-S5	22	4	0 (80)
	B-S11	6	5	0 (30)

### Data Analysis

For statistical analysis of brood size and offspring sex ratio, all-male broods were excluded because they were likely produced by unmated females. We verified this assumption by testing ploidy levels for 45 males from 11 all-male broods of the 5^th^ generation of B-S cross in *A. pleuralis* (on average four males were randomly sampled from each brood), and no diploid males were recorded for any of these, making it highly unlikely that these all-male broods are caused by homozygosity at all *csd* loci. To account for the variation of genetic relatedness among different types of crosses, we used the coefficient of co-ancestry as an explanatory variable in data analysis. Coefficient of co-ancestry values, adjusted for haplodiploids, are 0 for an outcross; 0.5 for a M-S cross, 0.5, 0.625, 0.688, 0.750, 0.797, 0.836, 0.867, 0.893, 0.913, 0.930, 0.943 and 0.954 for up to 12 successive generations of B-S crosses respectively [44].

Data from the different types of crosses were compared using generalized linear models (glm) to account for the appropriate error structure. Brood size, number of male and female offspring are non-normally distributed count data and were analyzed using a log link function and a quasi-poisson error structure to correct for overdispersion. In the brood size glm analysis, brood size, male and female offspring were used as the response variable and the coefficient of co-ancestry as explanatory variable. Offspring sex ratio data are proportional and were analyzed using a logit link function and a quasi-binomial error structure to correct for overdispersion. In the sex ratio glm analysis, the number of males was used as the response variable, brood size as the binomial denominator, and the coefficient of co-ancestry as explanatory variable. In the pupal mortality glm analysis, the number of black pupae was used as the response variable, total pupae as the binomial denominator, and the coefficient of co-ancestry as explanatory variable. All statistical analyses were performed with R 2.13.0 [45], comparisons of traits among generations were done using the R package multcomp [46].

### Data Simulations

De Boer et al. [25]–[26] developed individual-based simulation models to compare and statistically test the observed and predicted proportion diploid males (proportion diploid males among diploid offspring) and offspring sex ratios under CSD with a maximum of three *csd* loci. Cook [24] stated that ml-CSD can be strongly rejected if a maximum of ten *csd* loci can be ruled out. In our study, individual-based simulations, similar to de Boer et al. [26], were performed with varying numbers of putative unlinked *csd* loci, *n*_loci_ (1, 2, 5 or 10) to compare the observed and predicted proportion diploid males and offspring sex ratios over successive generations of inbreeding. The model was set up to mimic our experiment, assuming the same number of female wasps in each generation for each species in our inbreeding experiment. A simulation was initiated by allowing females that are heterozygous at all *csd* loci to produce a number of haploid sons (*n*_hm_), from which one son was sampled that mated with the female (M-S cross). Subsequently, each mated female produced a number of diploid offspring (*n*_d_). The numbers *n*_hm_ and *n*_d_ were randomly drawn values from the overall distribution of diploid family sizes or sons produced by outbred females in our experiment. A given diploid offspring developed as a female, unless it was homozygous for all its *n*_loci_ *csd* loci, in which case it developed as a diploid male. Each diploid male was assumed to have similar survival as their female siblings, which was validated by our experimental data (see below). The pool of newborn females and haploid males produced by each mother was then used to initiate the subsequent generation of B-S crosses, in which the production of diploid and haploid offspring occurred in a similar fashion as in the previous generation. Linkage between loci would result in outcomes intermediate to the distinct loci numbers (results not shown). A detailed simulation model description is presented in the supporting information, Text S1.

## Results

### Detection of Diploid Males

Ploidy was analyzed using flow cytometry for a selected number of male offspring from the first three and the last generations of inbreeding for four tested *Asobara* species. On average, four, five or six males per brood (resulting in a total of 52, 47 and 67 male samples respectively) were randomly selected from the M-S cross of *A. tabida*, *A. citri* and *A. japonica* (Table 1). Not a single diploid male was detected. In *A. pleuralis*, one diploid male was detected among 147 males that were randomly selected from all 12 M-S broods (Table 1). In another sample, two to four males per brood were randomly selected from the 1^st^ and 2^nd^ generations of B-S crosses of each species (resulting in 80–98 males in each tested species), and no diploid males were detected except for a single one (among 48 males in total) in the 2^nd^ generation of B-S cross of *A. citri* (Table 1, Figure S1). Finally, five to six males per brood (resulting in 30–36 males) were randomly selected from the last or the second last generation of B-S cross of each species. No diploid males were detected (Table 1).

### Brood Size, Offspring Sex Ratio and Pupal Mortality Under Inbreeding

**Figure 1:**
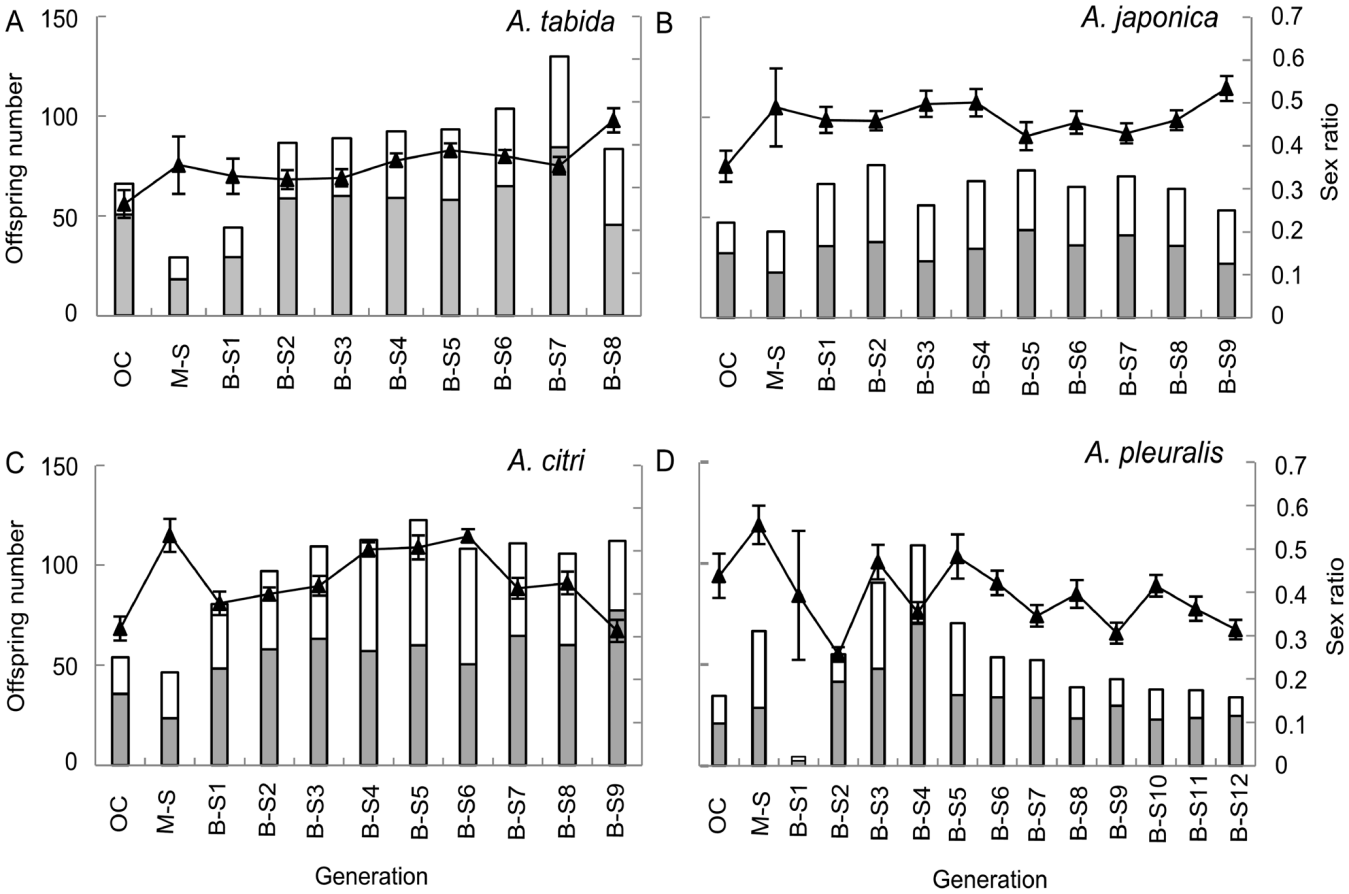
Secondary offspring sex ratio, brood size, male and female offspring numbers over generations of inbreeding. (a): *Asobara tabida*, (b): *A. japonica*, (c): *A. citri*, (d): *A. pleuralis*, OC: outcross. Open and grey bars denote male and female offspring number respectively. Black triangles represent mean sex ratio, and error bars represent standard error.

Though virtual absence of diploid males can be taken as strong evidence for absence of CSD, diploid males can also be inviable and would then go undetected. We therefore monitored the brood size, offspring sex ratio and pupal mortality in each generation. Offspring sex ratio is predicted to increase under CSD regardless of diploid male survival, since diploid male production is at the cost of females, although survival of diploid males leads to a stronger shift in sex ratio towards males [26], [27], [31]. Pupal mortality was low (typically only a few percent and rarely above 10%) over all generations of each tested *Asobara* species (Table S2). In *A. tabida*, one generation of outcross was followed by one generation of M-S cross and eight generations of B-S crosses. Both male and female offspring numbers increased significantly (Figure 1A, males: glm *F*_1, 387_ = 131.15, *P*<0.0001; females: glm *F*_1, 387_ = 29.18, *p*<0.0001). Brood size of inbreeding crosses was overall approximately 20% larger than of outcross, except for the initial M-S cross and the 1^st^ generation of B-S cross (Figure 1A, glm *F*_1, 387_ = 106.10, *p*<0.0001). In addition, the proportion pupal mortality of inbreeding crosses was significantly lower than of the outcross, except for the M-S cross (glm *F*_1, 173_ = 13.78, *p*<0.0001). As we offered the same number of 150 host larvae, these results indicate that there is no larva-to-adult wasp mortality due to inviable diploid males. In addition, offspring sex ratio was slightly (approximately 5% overall) but significantly increasing over the generations of inbreeding (Figure 1A, glm *F*_1, 387_ = 30.35, *p*<0.0001; Table S2).

For *A. japonica* and *A. citri*, one generation of outcross was followed by one generation of M-S cross and nine generations of B-S crosses. In *A. japonica*, male and female offspring numbers as well as brood size did not change over all generations (Figure 1B, males: glm *F*_1, 344_ = 1.19, *p* = 0.28; females: glm *F*_1, 344_ = 1.23, *p* = 0.27; brood size: glm *F*_1, 344_ = 2.10, *p* = 0.15). In addition, the proportion pupal mortality of multiple inbreeding generations was not higher than of the outcross (Table S2, glm *F*_1, 181_ = 1.36, *p* = 0.24). In *A. citri*, brood size as well as both male and female offspring numbers increased significantly over inbreeding generations (Figure 1C, brood size: glm *F*_1, 413_ = 187.30, *p*<0.0001; males: glm *F*_1, 413_ = 84.08, *P*<0.0001; females: glm *F*_1, 413_ = 69.54, *p*<0.0001). The proportion pupal mortality of inbreeding generations was not higher than of the outcross, with the exception of a slight increase in the M-S cross (Table S2, glm *F*_1, 184_ = 6.72, *p* = 0.01). These observations again indicate that no larva-to-adult wasp mortality is due to diploid male mortality in these two species. Furthermore, compared to outcrosses, the offspring sex ratios did not change over all successive generations for both *A. japonica* (Figure 1B, glm *F*_1, 344_ = 0.17, *p* = 0.68) and *A. citri* (Figure 1C, glm *F*_1, 413_ = 3.42, *p* = 0.07; Table S2).

For *A. pleuralis*, twelve generations of B-S crosses were performed after one generation of random mating from the mass culture and one generation of M-S cross. No directional patterns were observed in both male offspring number and brood size over inbreeding generations: it increased in the first several generations and decreased in later generations but did not deviate from the outcross (Figure 1D, male offspring: glm *F*_1, 457_ = 8.59, *P* = 0.004; brood size: glm *F*_1, 457_ = 6.22, *p* = 0.013). The number of female offspring, however, did not change significantly over generations (Figure 1D, glm *F*_1, 457_ = 1.36, *p* = 0.24). The offspring sex ratio fluctuated among successive generations of inbreeding, but overall was not higher than the outcross (Figure 1D, glm *F*_1, 457_ = 7.47, *p* = 0.007). Unfortunately, no data were obtained for pupal mortality of the M-S cross and the outcross in this species. The pupal mortality in inbreeding generations, however, showed a constantly low proportion (8.5% on average, Table S2, glm *F*_1, 116_ = 1.51, *p* = 0.22). Again, larva-to-adult wasp mortality was not prominent in this species. The low brood size observed in the first generation of B-S cross in *A. pleuralis* (Figure 1D) resulted from a rearing problem in the experiment: only daughters of old age (5 weeks at 12°C) were available from the M-S cross to set up the next generation. In addition, offspring sex ratio overall decreased significantly over successive inbreeding generations (Figure 1D, glm *F*_1, 457_ = 7.47, *p* = 0.007), which is opposite to the prediction under CSD.

### Diploid Male and Offspring Sex Ratio Compared with Simulations

**Figure 2:**
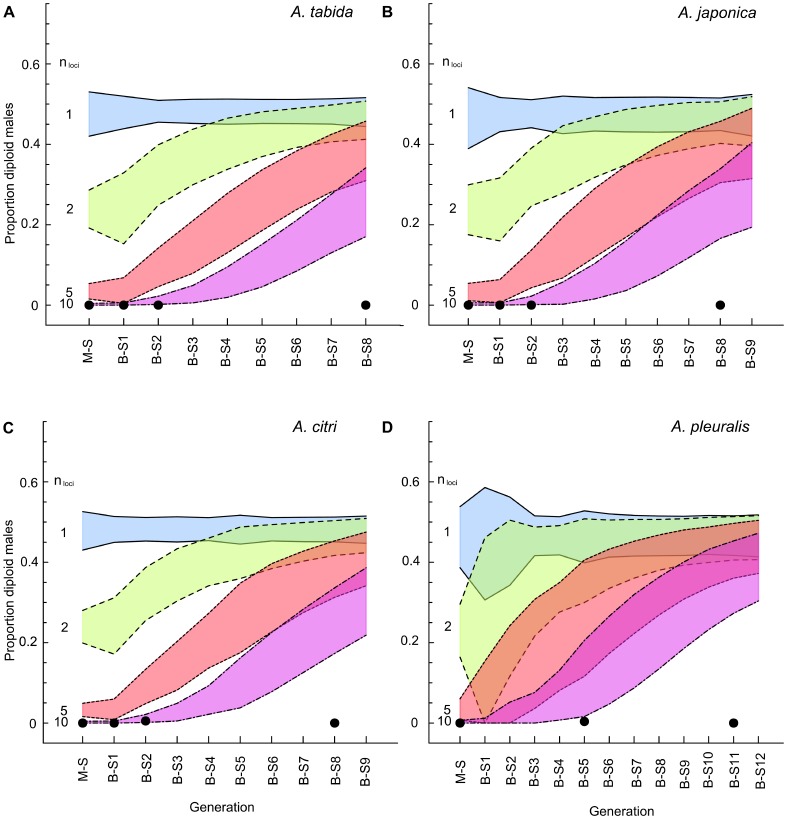
Simulation of the proportion diploid males. (a): *Asobara tabida*, (b): *A. japonica*, (c): *A. citri* and (d): *A. pleuralis*. 10 000 replicates of each experiment were simulated, assuming different numbers of unlinked *csd* loci, *n*_loci_ = {1, 2, 5 and 10}. Blue shading with solid lines represent predicted proportion diploid males under CSD with one locus; green shading with dashed lines represent the trend under CSD with two loci; red shading with dotted lines for five loci, and pink shading with dot-dashed lines for ten loci. Each shaded polygon represents the 95% confidence intervals of the proportion diploid males for a particular number of *csd* loci. Black dots are the observed proportion diploid males in our experiments.

Under CSD with a single locus, simulations predicted a stable proportion of diploid males for all tested species (around 0.5) over successive generations of inbreeding (Figure 2). Under CSD with two, five or ten unlinked loci, a gradual increase in the proportion diploid males towards 0.5 was predicted (Figure 2). In contrast to these predictions, no diploid males were found in *A. tabida* and *A. japonica* over nine or ten inbreeding generations respectively (Figure 2A and B), and only a single diploid male was found in *A. citri* (during the 2^nd^ generation of the B-S cross) and *A. pleuralis* (during the M-S cross) (Table1, Figure 2C and D). The lack of a progressive increase in the number of diploid males across all species is inconsistent with model predictions for all tested species for ml-CSD with up to ten loci.

**Figure 3:**
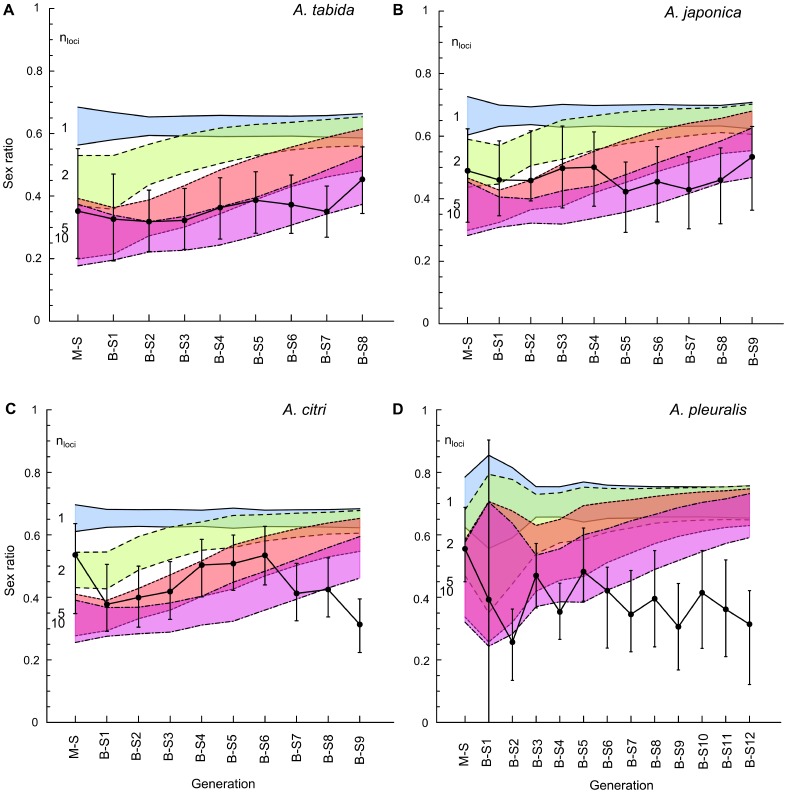
Simulation of secondary offspring sex ratios. (a): *Asobara tabida*, (b): *A. japonica*, (c): *A. citri* and (d): *A. pleuralis*. 10 000 replicates of each experiment were simulated, assuming different numbers of unlinked *csd* loci, *n*_loci_ = {1, 2, 5 and 10}. Blue shading with solid line represents predicted offspring sex ratio under CSD with one locus; green shading with dashed line represents the trend under CSD with two loci; red shading with dotted line for five loci, and pink shading with dot-dashed line for ten loci. Each color-shaded polygon represents the 95% confidence intervals of offspring sex ratio for a particular number of *csd* loci *n*_loci_, which is listed on the left side of the polygons. Black dots represent observed mean offspring sex ratio per generation, and corresponding error bars represent 95% confidence intervals of the observed mean offspring sex ratio. Note that in *A. pleuralis*, the low initial brood size (on average five) in the 1^st^ generation of the B-S cross makes the stochastic effects more pronounced, resulting in overlapping confidence intervals for model predictions during the first generations (the same effect also occurs in simulations for proportions of diploid males in Figure 2.).

The simulations for offspring sex ratios confirm the predictions for proportions diploid males, and predict that offspring sex ratios should approach approximately 0.65 under sl-CSD, and converge towards similarly high values under ml-CSD with two, five, or ten loci over multiple inbreeding generations. Compared to the outcross experiments, offspring sex ratios vary only slightly within the range of 0.30–0.55 for *A. tabida* and *A. pleuralis* (Figure 3A and D), or remained unchanged around 0.45 for *A. japonica* and *A. citri* (Figure 3B and C). Comparison of the empirical data to the simulations indicates that sl-CSD is absent in all tested *Asobara* species (Figure 3). Ml-CSD with up to at least five loci can also be ruled out in all species, because observed offspring sex ratios remained consistently lower than the range of predicted 95% confidence intervals for ml-CSD with five loci, and there was no dramatic increase in sex ratio over progressive generations of inbreeding. Moreover, for *A. citri* and *A. pleuralis*, ml-CSD with up to ten loci can be rejected, because the observed sex ratios over successive generations of inbreeding were below the predicted 95% confidence intervals for CSD with up to ten loci, and sex ratios decreased rather than increased over progressive generations of inbreeding. The observed offspring sex ratios in *A. tabida* and *A. japonica*, however, did not allow us to exclude ml-CSD with ten loci (Figure 3). Comparing our experimental results with simulations thus suggests that, if present, ml-CSD should consist of a substantial number of loci in all species (at least five in *A. tabida* and *A. japonica*, and more than ten in *A. citri* and *A. pleuralis* (Figure 3)). Or, as is deemed more likely, CSD is absent altogether in this group of species.

## Discussion

In this study, we found no diploid males (with the exception of two individuals), no decreased brood sizes, no substantially increased offspring sex ratios, and no increased pupal mortality over successive generations of strict inbreeding in four tested *Asobara* species, indicating that another mechanism than CSD is underlying sex determination in these species. Absence of diploid males is crucial but no conclusive evidence for absence of CSD, because diploid males could be inviable [23], [27], [47]. If diploid males do not survive, both female offspring number and brood size are expected to decrease over inbreeding generations, since diploid male production comes at the cost of female production under CSD. In addition, offspring sex ratio is expected to gradually increase due to loss of sex alleles. Our data, however, do not show these predicted patterns (Figure 1, Table S2). Only for *A. tabida*, a slight increase was observed in offspring sex ratio during progressive inbreeding. As female offspring numbers also increased and pupal mortality decreased simultaneously, the most likely explanation for this pattern is purging of a genetic load in early inbreeding generations, perhaps combined with outbreeding depression [48] in the first outcross generation. The single diploid male each in broods of *A. citri* and *A. pleuralis* are likely the result of a rare genetic mutation or an endoduplication event rather than from matched *csd* alleles. Endoduplication during early development after sex determination may yield diploid tissues in males as is known for muscle cells in the Hymenoptera [49]. Occasional diploid males have been found in other non-CSD parasitoids [29], [50]. In conclusion, there are no indications for diploid male mortality in all four tested *Asobara* species.

With the confirmed assumption of no diploid male mortality, the simulation models allowed us to rule out CSD involving up to ten loci for all tested *Asobara* species, though sex ratio simulations could not rule out ten loci for *A. tabida* and *A. japonica*. Cook [24] stated that rejection of CSD involving up to ten loci is a strong refutation of the ml-CSD model, since selection maintaining polymorphism at each sex locus is weaker and therefore limits the number of functional loci [14], [51]. He further argued that ten generations of inbreeding is more than adequate to test for CSD involving up to 15 loci [24]. Following this reasoning, we can safely reject both sl-CSD and ml-CSD in all tested *Asobara* species.

CSD is considered to be incompatible with Local Mate Competition (LMC) [21]–[23], [27], [29], which occurs in subdivided populations when brothers compete to mate with their sisters [52]. The reason is that LMC would dramatically decrease fitness due to diploid male production upon inbreeding. Some degree of LMC occurs in *Asobara* due to the patchy and aggregated distribution of their hosts (W. Ma et al., unpublished data). In addition, specific mating behaviors may contribute to different inbreeding levels among the four tested *Asobara* species. Females of *A. tabida* [35] and *A. japonica* (W. Ma et al., unpublished data) often aggregate during host-searching behavior, and a certain level of outcrossing likely occurs among offspring from multiple non genetically related females. In *A. citri,* the mating structure is strongly affected by female patch defense behavior [35]. Patch defense behavior is expected to increase the inbreeding level, because in most cases only a single female monopolizes the host patch [35], which will intensify LMC. The mating structure of *A. pleuralis* is less well studied. Ml-CSD is one way to reduce the fitness cost due to diploid males, and in different *Cotesia* species with diverse inbreeding levels there appears to be a link between mating system and absence or presence of different types of CSD [25], [26], [28], [30]–[32]. We do not see such an association in the genus *Asobara*, which could be due to phylogenetic constrains or other reasons. Taken together, the absence of CSD in the four tested *Asobara* species is consistent with the limited information available on the inbreeding levels in natural populations.

Sl-CSD has been demonstrated in species from each major hymenopteran subgroup, including sawflies (Symphyta), parasitoid wasps (Apocrita; Parasitica), and ants, bees and wasps (Apocrita; Aculeata) [9], [18]. As an alternative mechanism to sl-CSD, ml-CSD has been proposed to evolve from sl-CSD by one or more duplications of the sex locus [19], [20], or through tandem or segmental duplication of the *csd* gene [53], [54]. It has so far only been documented in two *Cotesia* species [25], [26], and multiple *csd* genes have yet to be identified in any species. It is still under debate whether sl-CSD is the ancestral mode of sex determination, and more species need to be tested to reach a firm conclusion about the phylogenetic distribution of CSD in the Hymenoptera [9], [18]–[20]. CSD has been ruled out in many chalcidoid and cynipoid wasps [9], [10]. Our results add four species lacking CSD to the family of Braconidae, which has previously been reported to contain both species with and without CSD [8], [9], [25], [26], [28], [30]–[32]. Our results also reject Asplen et al.’s hypothesis [18] of ml-CSD in *Asobara*, and further calls for a new phylogenetic reconstruction of CSD in the Hymenoptera. Even though several alternative mechanisms have been proposed over the years (reviewed in [11]), the alternatives to CSD in the Hymenoptera are poorly understood. The only other empirically supported sex determination mechanism is maternal control sex determination in *Nasonia vitripennis*. In contrast to CSD, this mechanism operates independently of inbreeding levels consistent with a highly subdivided population structure and associated strong LMC in this species [11], [16], [17], [42]. For the moment maternal control sex determination could be a potential candidate mechanism for the *Asobara* genus. The bottleneck for elucidating the exact sex determining mechanism in *Asobara* and other hymenopteran genera is a lack of detailed genome information. However, with the current developments in next-generation sequencing technologies, this information gap may soon be closed.

## Supporting Information

Figure S1 **Flow cytometric DNA-histograms of a representative diploid female (a), diploid male (b) and haploid male (c) in** ***A. citri*** **.** On the y axis is the number of nuclei, and the x axis is the fluorescence intensity in a log scale, which converts to ploidy in this figure. An excitation wave length of 488 nm and a band pass filter of 585 nm were used to detect propidium iodide fluorescence. 2500 nuclei were measured in each sample in an FL2-W/FL2-A gated region containing haploid and diploid cells. The small diploid peaks in these figures represent an endoduplication in some tissues as is typical for haploid hymenopterans [49].
(PDF)

Table S1 **Collection sites and rearing conditions of the four** ***Asobara*** **species used in this study.**
(DOC)

Table S2 **Comparison of sex ratio (SR), brood size (BS) and pupal mortality (PM) between outcrosses and multiple generations of inbreeding in** ***Asobara tabida, A. japonica, A. citri*** **and** ***A. pleuralis*** **.**
(DOCX)

Text S1 **Individual-based simulations.**
(DOCX)
