# Supplementary figures and images for: Absence of Complementary Sex Determination in the Parasitoid Wasp Genus Asobara (Hymenoptera: Braconidae)
Source: PLoS One. 2013 Apr 2;8(4):e60459. doi: 10.1371/journal.pone.0060459 (PMC3614920; doi:10.1371/journal.pone.0060459)

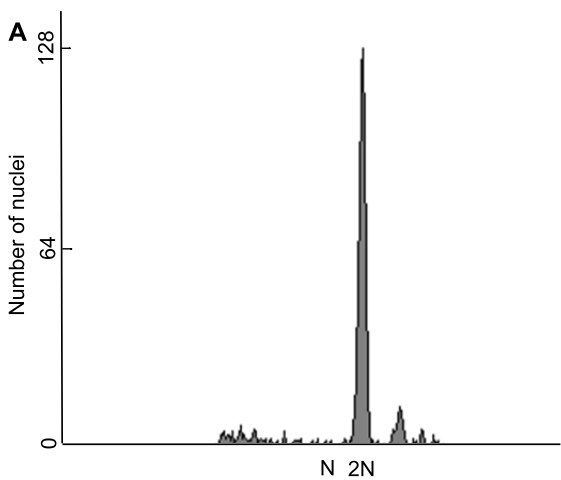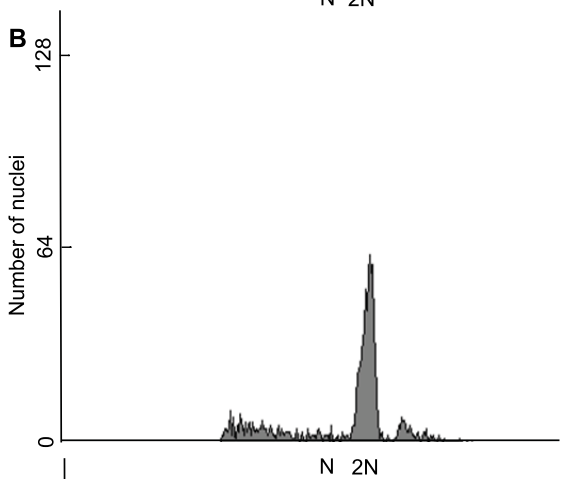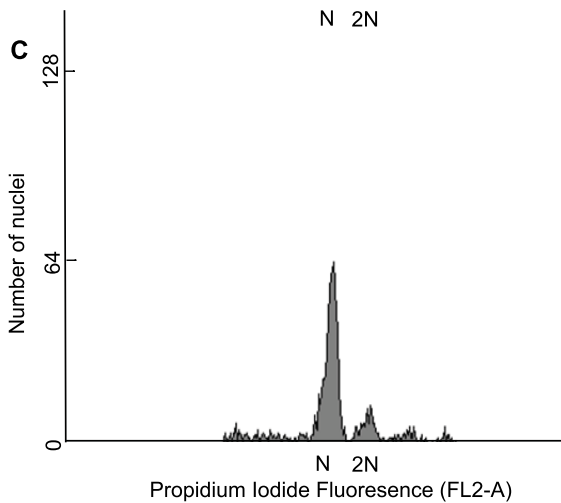

**Figure S1**

Supplement: Figure S1 — Flow cytometric DNA-histograms of a representative diploid female (a), diploid male (b) and haploid male (c) in A. citri . On the y axis is the number of nuclei, and the x axis is the fluorescence intensity in a log scale, which converts to ploidy in this figure. An excitation wave length of 488 nm and a band pass filter of 585 nm were used to detect propidium iodide fluorescence. 2500 nuclei were measured in each sample in an FL2-W/FL2-A gated region containing haploid and diploid cells. The small diploid peaks in these figures represent an endoduplication in some tissues as is typical for haploid hymenopterans [49]. (PDF) [file pone.0060459.s001.pdf]
