# Supplementary material for: Absence of Complementary Sex Determination in the Parasitoid Wasp Genus Asobara (Hymenoptera: Braconidae)
Source: PLoS One. 2013 Apr 2;8(4):e60459. doi: 10.1371/journal.pone.0060459 (PMC3614920; doi:10.1371/journal.pone.0060459)
Supplement: Table S1 — Collection sites and rearing conditions of the four Asobara species used in this study. (DOC) [file pone.0060459.s002.doc]

| **Table S1.** | | | | | |
| --- | --- | --- | --- | --- | --- |
| **Species** | **S Strain** | **Collection site** | **Habitat** | **Host species** | **Rearing temperature** |
| ***A. tabida*** | Sos | Sospel, France | Temperate and wet | *D. subobscura* | 20°C |
|  | Italy | Pisa, Italy |  | *D. melanogaster* | 20°C |
| ***A. japonica*** | Am | Amami-oshima,  Japan | Temperate and wet | *D. melanogaster* | 25°C |
|  | Irio | Iriomote-jima,  Japan |  | *D. melanogaster* | 25°C |
| ***A. citri*** | Ivory | Lamto,  Côte d’Ivoire | Hot with alternating  dry and wet seasons | *D. melanogaster* | 25°C |
| ***A. pleuralis*** | Manado | Manado, Sulawesi,  Indonesia | Tropical wet forest | *D. melanogaster* | 25°C |
