# Supplementary material for: Absence of Complementary Sex Determination in the Parasitoid Wasp Genus Asobara (Hymenoptera: Braconidae)
Source: PLoS One. 2013 Apr 2;8(4):e60459. doi: 10.1371/journal.pone.0060459 (PMC3614920; doi:10.1371/journal.pone.0060459)
Supplement: Table S2 — Comparison of sex ratio (SR), brood size (BS) and pupal mortality (PM) between outcrosses and multiple generations of inbreeding in Asobara tabida, A. japonica, A. citri and A. pleuralis . (DOCX) [file pone.0060459.s003.docx]

| **Table S2.** | | | | | | | | | | | | | | | | | | |  |  |
| --- | --- | --- | --- | --- | --- | --- | --- | --- | --- | --- | --- | --- | --- | --- | --- | --- | --- | --- | --- | --- |
| **GEN^1^** | 1. ***tabida*** | | | | 1. ***japonica*** | | | | 1. ***citri*** | | | | 1. ***pleuralis*** | | | | | | |  |
|  | **REP^2^** | **SR**  **(%)** | **BS** | **PM**  **(%)** | **REP** | **SR**  **(%)** | **BS** | **PM**  **(%)** | **REP** | **SR**  **(%)** | **BS** | **PM**  **(%)** | **REP** | | **SR**  **(%)** | **BS** | **PM**  **(%)** | |  |  |
| **OC^3^** | 31 | 26.1±3.2 | 66±4 | 21.5±2.1 | 14 | 35.3±3.7 | 47±4 | 8.4±1.2 | 26 | 31.9±2.8 | 54±4 | 4.6±0.6 | 21 | 43.8±5.1 | | 35±4 | | - | | |
| **M-S** | 12 | 35.2±6.7 | 29±5 | 35.0±3.3 | 9 | 48.9±9.1 | 43±8 | 11.7±1.5 | 16 | 53.6±3.9 | 47±4 | 13.2±1.0 | 12 | 55.5±4.4 | | 66±5 | | - | | |
| **B-S1** | 25 | 32.7±4.1 | 44±4 | 12.9±2.0 | 34 | 46.0±3.0 | 67±4 | 4.0±0.5 | 38 | 37.8±2.7 | 80±6 | - | 4 | 39.2±14.9 | | 5±1 | | - | | |
| **B-S2** | 57 | 31.8±2.3 | 87±3 | 9.5 ±0.7 | 52 | 45.8±2.3 | 76±8 | - | 46 | 40.0±1.5 | 97±2 | - | 7 | 25.7±1.7 | | 55±8 | | 8.3±1.3 | | |
| **B-S3** | 48 | 32.2±2.0 | 89±4 | - | 30 | 50.0±3.1 | 56±4 | 5.9±0.7 | 40 | 41.9±2.3 | 109±3 | 5.3±0.5 | 43 | 47.0±4.0 | | 90±4 | | - | | |
| **B-S4** | 43 | 36.3±1.7 | 92±3 | - | 38 | 50.1±3.2 | 68±3 | - | 49 | 50.3±1.6 | 113±4 | - | 45 | 35.4±2.3 | | 109±4 | | 10.8±0.8 | | |
| **B-S5** | 48 | 38.7±1.6 | 93±3 | 13.2±0.8 | 35 | 42.2±3.3 | 73±4 | 7.4±0.9 | 30 | 50.8±2.8 | 123±3 | 4.1±0.4 | 26 | 48.2±5.1 | | 71±6 | | 6.8±0.9 | | |
| **B-S6** | 48 | 37.3±1.5 | 104±2 | - | 34 | 45.4±2.7 | 65±3 | - | 47 | 53.5±1.6 | 108±2 | - | 38 | 42.1±2.8 | | 54±5 | | - | | |
| **B-S7** | 43 | 35.1±2.0 | 130±3 | - | 36 | 42.9±2.3 | 71±2.5 | 6.9±0.4 | 45 | 41.3±2.4 | 111±5 | - | 41 | 34.6±2.5 | | 52±5 | | -  - | | |
| **B-S8** | 33 | 45.7±2.8 | 84±4 | 18.0±1.1 | 39 | 46.0±2.3 | 64±4 | - | 42 | 42.5±2.7 | 106±3 | - | 46 | 40.0±3.2 | | 39±4 | |  | | |
| **B-S9** | - | - | - | - | 24 | 53.4±2.9 | 54±6 | 8.9±0.5 | 35 | 31.3±2.6 | 112±2 | 4.8±0.5 | 45 | 30.6±2.4 | | 43±5 | | - | | |
| **B-S10** | - | - | - | - | - | - | - | - | - | - | - | - | 45 | 41.5±2.5 | | 38±5 | | - | | |
| **B-S11** | - | - | - | - | - | - | - | - | - | - | - | - | 45 | 36.2±2.8 | | 37±4 | | - | | |
| **B-S12** | - | - | - | - | - | - | - | - | - | - | - | - | 40 | 31.4±2.3 | | 34±5 | | 9.3±0.5 | | |
| ^1^ generation (GEN)  ^2^ replicate (REP)  ^3^ outcross (OC) | | | | | | | | | | | | | | | | | | | | |
