# Supplementary material for: Absence of Complementary Sex Determination in the Parasitoid Wasp Genus Asobara (Hymenoptera: Braconidae)
Source: PLoS One. 2013 Apr 2;8(4):e60459. doi: 10.1371/journal.pone.0060459 (PMC3614920; doi:10.1371/journal.pone.0060459)
Supplement: Text S1 — Individual-based simulations. (DOCX) [file pone.0060459.s004.docx]

**Text S1.**

Individual-based simulations were used to mimic the actual inbreeding experiments, allowing comparison of observed and predicted diploid male proportions and offspring sex ratios over the course of the experiment for different numbers of unlinked *csd* loci, *n*_loci_ (1, 2, 5 or 10). A simulation run started with a number of M-S crosses, identical to the number of M-S crosses in the actual experiment. Mothers were assumed to be heterozygous for all of their putative *csd* loci, which resulted in conservative predictions of proportion diploid males and offspring sex ratios. M-S crosses were simulated by allowing the mother to produce a single haploid male that mates with her, after which mothers produce haploid males and diploid offspring. Diploid offspring develop as males only when *csd* alleles at all sex loci are identical. The total number of diploid offspring produced by any mother is a randomly sampled value from the overall distribution of diploid offspring produced by outbred females in our experiment. For each simulated M-S cross, the number of diploid sons (*n*_dm_) was then recorded. Diploid males are assumed to have the same developmental survival rate as diploid females (*n*_f_). Each mother also produced a number of haploid sons (*n*_hm_), where *n*_hm_ was a randomly drawn value from the overall distribution of sons produced by outbred females.

Each generation of B-S crosses was simulated in the same fashion as the mother-son cross. One to three virgin females were randomly sampled per family from the previous generation. Each selected female mated with a haploid male that was sampled from her *n*_hm_ haploid brothers. As in the M-S cross, the total number of diploid offspring produced by any mother is a randomly sampled value from the distribution of diploid offspring produced by outbred females. Again, the number of diploid sons (*n*_dm_) was recorded and each mother also produced a number of haploid sons (*n*_hm_), where *n*_hm_ was a randomly drawn value from the overall distribution of sons produced by outbred females. These B-S crosses were subsequently repeated for the same number of generations as in the actual experiment.

Offspring sex ratios were predicted based on the prediction of diploid male numbers (from the simulations described above) and a number of haploid males (*n*_hm_)*,* a randomly drawn value from the overall distribution of sons produced by outbred females. The offspring sex ratio was calculated as (*n*_hm_*+n*_dm_)/(*n*_f_*+n*_hm_+*n*_dm_). This assumes that the production of haploid sons is unaffected by inbreeding level and a mother’s investment in diploid offspring. Simulations were replicated 10 000 times.
